# Could social relationships be key to reaching healthy longevity?

**DOI:** 10.18632/aging.204871

**Published:** 2023-06-29

**Authors:** Antonio Garrido, Mónica De la Fuente

**Affiliations:** 1 Department of Health Sciences, Faculty of Biomedical and Health Sciences, Universidad Europea de Madrid, 28670 Madrid, SpainSpain; 2 Department of Genetics, Physiology, and Microbiology (Animal Physiology Unit), Faculty of Biology, Complutense University of Madrid, 28040 Madrid, Spain; 3 Institute of Investigation 12 de Octubre (i+12), 28041 Madrid, Spain

**Keywords:** social environment, aging strategy, immunosenescence

Although there are still unanswered questions about the biological mechanisms governing how social relationships can be fundamental to reaching healthy aging, nowadays, growing evidence indicates that the maintenance of these positive social bounds triggers the slow-down of aging consequences and potentiates the health span. It is well-known that health depends on the genome of each individual but to a greater extent on his/her “ambiome” (the environment in which an individual grows as well as the lifestyle that he/she follows in his/her adult life). In this context, in recent years, a series of studies have shown the relevance of social relationships by improving the functional response of physiological systems, especially those involved in the general homeostatic control, such as nervous, endocrine, and immune systems (reviewed in [1]). Thus, social interactions are a fundamental and adaptative component of the biology of social species, such as rodents and humans, which need to develop these bounds to carry out important survival functions, such as reproduction. In fact, the increase in human life expectancy that occurred during the twentieth century was favored by the development of vaccines and the use of antibiotics but also influenced by social improvements (health care, housing, and education). Consequently, a regression of the demographic pyramid appeared by the increase in the world´s population over 60 years old. This effect together with the early start of the aging process in humans (at adult age; in their twenties) explains the growing number of studies of aging research, demonstrating the need to ensure the best conditions to carry out this process, and increasing, therefore, the health span of the individual.

It is known that aging is a multifaceted process characterized by a progressive and general decline of the organism´s functions. This impairment affects all physiological systems, but the immune system is especially compromised. The immune deterioration associated with aging involves an evident rearrangement of almost every immune component, triggering enhanced and diminished immune functions, a process known as immunosenescence. This age-related loss of immune competence is clearly shown by the high susceptibility to infections and mortality, as well as the increase in the incidence of autoimmune diseases and cancer observed in aged individuals. Among the aging-related immune changes, a general impairment of T lymphocyte functions, such as lymphoproliferative responses to antigens, appears, but the innate immune responses are also affected, involving inadequate functions of phagocytes (macrophages, neutrophils, and dendritic cells) and *Natural Killer*, cells. Consequently, an imbalance of cytokine networks occurs in aging. A pro-inflammatory cytokine profile in absence of antigenic stimuli is established in aged individuals, arising the sterile inflammation, characteristic of aging. This promotes low pro-inflammatory cytokine release by immune cells from old individuals as a response to an antigenic or mitogenic stimulus. Altogether, this immunosenescence, as we suggested many years ago, can be implicated in “oxi-inflamm-aging” and so, in the aging rate of each individual and consequently in his/her longevity (reviewed in [2]).

In this regard, several lifestyle strategies, such as nutrition and exercise, have been extensively proposed as potential interventions able to preserve an adequate immune response and thus, maintain health and thereby slow down the aging rate. Similar effects have been revealed using social strategies in rodents. Thus, when chronologically old mice (84 weeks of age) cohabited with adult mice (37 weeks of age), the former exhibited a slow-down in their immunosenescence, thereby improving several immune functions compared to chronologically old mice not exposed to this social strategy. Consequently, a longer lifespan was observed in the group subjected to social manipulation [3]. Interestingly, the beneficial effects of this social strategy not only are observed in chronological aging but also in adults with premature aging. In fact, prematurely aging mice (PAM) are characterized by a general impairment of immune function at chronologically adult age (37 weeks of age), exhibiting a premature immunosenescence and therefore a shorter lifespan compared to their exceptional non-prematurely aging counterparts (ENPAM). However, when these PAM live for two months with ENPAM, their immune functions are improved, reaching some of their similar values to those observed in ENPAM did not subject to this social manipulation. Also, their lifespan is positively affected [4, 5]. These beneficial effects of cohabitation were also analyzed in a genetic model of premature aging. Chronologically adult mice with the deletion of a single tyrosine hydroxylase (TH-HZ) allele, the limiting-rate enzyme of catecholamine (CA) synthesis, show a premature immunosenescence establishment due to the low CA content, which is translated into a shorter lifespan compared to their wild-type counterparts at the same age. However, if these TH-HZ animals cohabit with wild-type mice at adult age, their impairments are ameliorated, reaching in some immune functions similar values to those observed in wild-type counterparts [6]. Considering that the age-related changes in the immune functions show similar evolution in rodents and humans, these findings of the beneficial effects of social relationships improving the immune response and slowing down the aging consequences could be transferred to human beings.

Finally, an important question arises from these studies: *Why these social relationships are important to reaching healthy aging?* Although the reasons for this fact are still unknown, one possibility could be the necessity to generate anti-inflammatory-antioxidant mechanisms in species such as *Homo sapiens*. It is important to consider that we are descendants of those humans that were able to overcome the reproductive age by their great immune activity (those with low immune response, especially respect the production of inflammatory defenses against infections, died without descent) [7]. This high capacity for generating inflammation, which is associated with oxidation, is at the base of many diseases and the aging process. For this reason, the ingest of antioxidant diet (such as Mediterranean ones) or doing adequate exercise increases our antioxidant and anti-inflammatory reserves, and promotes healthy aging. Strikingly, social relationships can exert similar effects. In fact, chronologically old mice as well as chronologically adult PAM and TH-HZ mice after living for two months with chronologically adult mice exhibit a recovery of cytokine balance (low pro-inflammatory cytokines in absence of antigenic stimuli and adequate anti/pro-inflammatory cytokine release in presence of stimuli) compared to their counterparts not exposed to this social cohabitation [8]. Therefore, in social species such as humans, the maintenance of these social relationships could be understood as a health and survival tool, allowing an adequate immune response, which slows down the rate of aging. This would help us achieve healthy longevity.

In conclusion, considering the high percentage of older people around the world and the social and economic repercussions that this entails for any country, it is important to develop new lifestyle strategies to palliate the consequences of the aging process. In this sense, positive social relationships could be considered as an excellent way to slow down the aging rate and consequently increase the lifespan, and more importantly, healthy longevity, which would be of advantage to people and reduce the economic and social impact of aging in our societies.
